# ATR kinase activation in G1 phase facilitates the repair of ionizing radiation-induced DNA damage

**DOI:** 10.1093/nar/gkt833

**Published:** 2013-09-14

**Authors:** Armin M. Gamper, Reza Rofougaran, Simon C. Watkins, Joel S. Greenberger, Jan H. Beumer, Christopher J. Bakkenist

**Affiliations:** ^1^Department of Radiation Oncology, University of Pittsburgh School of Medicine, Pittsburgh, PA, USA, ^2^Department of Cell Biology and Physiology, Center for Biologic Imaging, University of Pittsburgh School of Medicine, Pittsburgh, PA, USA, ^3^Department of Pharmaceutical Sciences, University of Pittsburgh School of Pharmacy, Pittsburgh, PA, USA and ^4^Department of Pharmacology and Chemical Biology, University of Pittsburgh School of Medicine, Pittsburgh, PA, USA

## Abstract

The kinase ATR is activated by RPA-coated single-stranded DNA generated at aberrant replicative structures and resected double strand breaks. While many hundred candidate ATR substrates have been identified, the essential role of ATR in the replicative stress response has impeded the study of ATR kinase-dependent signalling. Using recently developed selective drugs, we show that ATR inhibition has a significantly more potent effect than ATM inhibition on ionizing radiation (IR)-mediated cell killing. Transient ATR inhibition for a short interval after IR has long-term consequences that include an accumulation of RPA foci and a total abrogation of Chk1 S345 phosphorylation. We show that ATR kinase activity in G1 phase cells is important for survival after IR and that ATR colocalizes with RPA in the absence of detectable RPA S4/8 phosphorylation. Our data reveal that, unexpectedly, ATR kinase inhibitors may be more potent cellular radiosensitizers than ATM kinase inhibitors, and that this is associated with a novel role for ATR in G1 phase cells.

## INTRODUCTION

Ataxia telangiectasia mutated (ATM) and the related kinase ATM- and Rad3-related (ATR) are principal signal transducers that mediate DNA damage signalling. While ATM is recruited to DNA double strand breaks (DSBs) by the Mre11, Rad50 and Nbs1 complex, ATR and its constitutive interacting partner ATRIP bind to replication protein A (RPA)-coated single-stranded DNA (ssDNA). ATR can then be further activated by direct interactions with DNA topoisomerase 2-binding protein 1 (TopBP1), which is recruited to ssDNA/double-stranded DNA junctions by the Rad9-Rad1-Hus1 (9-1-1) complex. Claspin-mediated phosphorylation of Chk1 kinase at serines 317 and 345 by ATR regulates Chk1 activity (1). Chk1 targets cell division cycle protein 25 (CDC25) for degradation by phosphorylation-dependent ubiquitination, thereby preventing the activation of cyclin-dependent kinases (CDKs). Thus, ATR/Chk1 signalling is initiated at structures containing ssDNA and a junction between ssDNA/double-stranded DNA, and this is associated with S and G2 phase cell cycle checkpoints in mammalian cells (2).

ATR-activating structures are present when replication stress causes DNA polymerase and helicase complexes to be uncoupled at a replication fork, during nucleotide excision repair, and during homology-directed recombination (HDR) repair. ATR is activated after ionizing radiation (IR), and this may be associated with the DNA end resection of DSBs that induces RPA-coated DNA prior to the formation of Rad51 filaments during HDR (3,4). Because HDR is most efficient between sister chromatids, previous studies on ATR activation after IR have focussed on S and G2 phase (5). Furthermore, it has been proposed that CtBP-interacting protein (CtIP) phosphorylation by CDK2 is required for DNA end resection and that this restricts ATR kinase activation and Chk1 signalling after IR to S and G2 phase (6,7). This premise is challenged, however, by the recent finding that CtIP is dispensable for Chk1 phosphorylation after treatment with camptothecin or IR (8).

Because ataxia telangiectasia patients, who typically express no ATM protein, are the most radiosensitive humans that have been identified (9), it has long been postulated that ATM kinase inhibitors will significantly increase the efficacy of targeted radiotherapy. In contrast to ATM and its downstream target Chk2, ATR and its downstream target Chk1 are essential genes in the mouse (10–13). Although it is known that overexpression of a kinase inactive ATR mutant causes increased sensitivity to several DNA-damaging agents (3,4), the lethality of ATR deletion has impeded the study of ATR kinase-dependent signalling after IR.

Here, we used a reverse chemical genetics approach to study ATR function. Selective and reversible ATR kinase inhibitors allowed us to investigate the consequences of transient ATR kinase inhibition in cells after IR. Surprisingly, ATR inhibition caused significantly more potent radiosensitization than ATM inhibition. Transient ATR inhibition in synchronized cells revealed a novel role of ATR in G1 phase and identified a short time interval after IR where ATR activity is critical for the repair of IR-induced damage and cell survival. ATR colocalized with RPA foci and was activated in irradiated G1 phase cells in the absence of RPA2 phosphorylation. Thus, ATR activation does not require extensive DNA end resection as previously postulated, indicating a potential mechanism of ATR activation in G1 phase cells in the absence of HDR.

## MATERIALS AND METHODS

### Reagents

ATM kinase inhibitor KU55933 (KuDOS Pharmaceuticals, now AstraZeneca) was used at final concentrations of 10 μM. ATR kinase inhibitors ETP-46464 and Vertex compound 45 were synthesized at the Medicinal Chemistry Shared Resource of the Ohio State University Comprehensive Cancer Center (Columbus, OH). ETP-46464 and Vertex compound 45 were used at a final concentration of 10 μM. Chk1 kinase inhibitor UCN-01 (U6508, Sigma-Aldrich) and CDK4/6 kinase inhibitor PD0332991 (S1116, Selleck Chemicals) were used at a final concentration of 100 nM. ATM, ATR, Chk1 and CDK4/6 kinase inhibitors were reconstituted in dimethyl sulfoxide. Apurinic/apyrimidinic endonuclease 1 (APE1) inhibitor NSC332395 (14) (gift from Dr. Barry Gold, University of Pittsburgh) was used at 400 ng/ml and α-amanitin (Sigma) at 50 μg/ml. Premo cdc10-dependent transcript 1-red fluorescent protein (Cdt1-RFP) virus was purchased from Invitrogen.

### Cell culture and irradiation

Dr. Jiri Lukas (University of Copenhagen) and Dr. Stephen Jackson (University of Cambridge) provided U2OS cells stably expressing green fluorescent protein (GFP)-tagged ATR or p53-binding protein 1 (53BP1). Dr. Jill Siegfried (University of Pittsburgh Cancer Institute) provided the lung cancer cells 201 T and 239 T (15). U2OS, Calu6, H460 and cell line authentication were purchased from the American Type Culture Collection. Cells were cultured in Basal Medium Eagle/1% glutamine (Life Technologies) or Dulbecco’s modified Eagle medium (DMEM) (Lonza) supplemented with 10% fetal bovine serum (Atlanta Biologicals), 100 U/ml penicillin and 100 µg/ml streptomycin (Lonza). For synchronization, cells were blocked in S phase with 2 mM thymidine for 24 h, released for 4 h and blocked in M phase with 100 ng/ml nocodazole. Cells were γ-irradiated in a Shepherd Mark I Model 68 [^137^Cs] irradiator (J.L. Shepherd & Associates) at a dose rate of 0.711 Gy/min.

### Immunoblotting and cell staining

Antisera against ATM phospho-S1981 (EP1890Y, Epitomics), generic ATM (A1106, Sigma-Aldrich), Chk1 phospho-S345 (#2348S, Cell Signaling Technology), generic Chk1 (#2360, Cell Signaling Technology), glyceraldehyde 3-phosphate dehydrogenase (GAPDH) (ab9483, Abcam), ATR (sc-1887, Santa Cruz), RPA2 (ab2175, Abcam), RPA2 phospho-S4/8 (A300-245 A, Bethyl), geminin (sc-13015, Santa Cruz), cyclin A (sc-596, Santa Cruz), cyclin D1 (sc-718, Santa Cruz), CDC25C phospho-S216 (#1190-1, Epitomics) and generic CDC25C (#4688, Cell Signaling Technology) were used in immunoblotting and/or cell staining. Whole cell extracts were prepared in lysis buffer: 50 mM Tris–HCl, pH 7.5, 150 mM NaCl, 50 mM NaF, 1% Tween-20, 0.5% NP40 and 1× protease inhibitor mixture (Roche Applied Science). Cleared cell extracts were resolved in either 3–8% Tris-Acetate or 4–12% Bis-Tris gels (Invitrogen). For cell staining, cells were fixed in 2% paraformaldehyde, permeabilized with 0.5% Triton X100 in phosphate buffered saline and saturated with bovine serum albumin. Cells were then incubated with primary antibodies for 2 h at room temperature, washed and incubated with secondary antibody for 1 h at 4°C in the dark. Dilution of primary antibodies was 1:400 for anti-RPA2, 1:600 for anti-cyclin A, 1:200 for anti-geminin and 1:200 for anti-cyclin D1. Dilution of secondary antibodies was 1:300 for anti-mouse-AlexaFluor488 and anti-mouse-AlexaFluor555 (both from Invitrogen) and 1:1000 for anti-rabbit-Cy3 (Jackson Immunoresearch).

### Confocal microscopy and statistical analysis

Fixed cells for foci analysis were imaged on an inverted confocal microscope (Nikon A1, Nikon) controlled by NIS Elements software. Z-stacks were taken to cover the entire thickness of the cell in a step size of 0.4 µm. The excitation wavelengths for GFP, 4′, 6-diamidino-2-phenylindole (DAPI) and RFP were 488, 405 and 543 nm, respectively. The signal was collected through a 1.40 60× oil immersion objective. For live cell imaging, cells were mounted on an environmentally controlled live cell chamber and monitored for 24 h on a Nikon A1 microscope with a 60× 1.49 NA total internal reflection fluorescence (TIRF) objective. Foci were counted in at least three independent experiments after reassembling Z-stacks. Error bars denote standard deviations. A Student’s *t*-test was done for a two-tailed distribution of two samples with unequal variances. The *, ** and *** denote *P*-values smaller than 0.1, 0.05 or 0.01, respectively. To evaluate colocalization of GFP-ATR and RPA, foci were examined in a single Z-stack.

### Clonogenic survival assays

Cells were seeded in 60 mm petri dishes and treated with inhibitor(s) and increasing doses of IR. Drug treatments were removed 4 h post IR or 75 min post IR in the case of 1 h treatment. Vehicle dimethyl sulfoxide served as control. After 10–12 days, depending on the cell line, colonies were stained with Giemsa solution. A colony was defined as a cluster of ≥50 cells, presumably having formed from a single cell. Three independent experiments were performed, each in triplicate. Error bars denote standard deviations derived from triplicates.

## RESULTS

### Inhibition of ATR leads to more potent radiosensitization than inhibition of ATM

**Figure 1.**
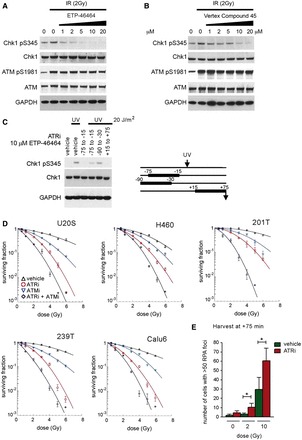
ATR inhibition has a more potent radiosensitizing effect than ATM inhibition. (**A** and **B**) ETP-46464 and Vertex Compound 45 are selective inhibitors of ATR. U2OS cells were treated with 2 Gy and the indicated concentration of ATR inhibitor. Immunoblots of lysates prepared 75 min following IR are shown for ETP- 46464 (A) and Vertex Compound 45 (B). (**C**) ETP-46464 is a reversible inhibitor of ATR. U2OS were treated with ETP-46464 before or after 20 J/m^2^ of ultraviolet light (UVC), as shown in the schematic on the right. Immunoblots of lysates prepared 75 min after irradiation are shown on the left. (**D**) Clonogenic survival assays of vehicle, KU55933 and/or ETP-46464 treated cells. Cell lines U2OS, H460, 201 T, 239 T and Calu6 were treated for 4.25 h (15 min prior to 4 h post IR) with 10 µM KU55933 and/or 10 µM ETP-46464. (**E**) ATR inhibition increases the number of IR-induced RPA foci at late time points. U2OS cells were treated with 0, 2 or 10 Gy IR and/or ETP-46464 for 4.25 h (15 min prior to 4 h post IR). Cells were fixed 24 h post IR and stained for RPA, and cells with >50 RPA foci were quantitated. Error bars denote standard deviations. 177 × 265 mm (300 × 300 DPI).

ETP-46464 (16) and Vertex compound 45 (17) are small molecules that selectively inhibit ATR kinase activity in cells. While 10 μM ETP-46464 inhibits the ATR-specific phosphorylation of Chk1 following 2 Gy of ionizing radiation (12,16), the auto-phosphorylation of ATM is not affected (Figure 1A and B). Furthermore, ATR kinase inhibition by ETP-46464 is rapidly reversible as removal of ETP-46464 just 30 min prior to ultraviolet irradiation restores the ability of ATR to phosphorylate Chk1 (Figure 1C). This selective and transient inhibition of ATR kinase activity has allowed the temporal inhibition of cellular ATR kinase activity while minimally affecting the survival of undamaged cells.

Selective small molecule ATM kinase inhibitors have been shown to radiosensitize cells *in vitro* (18). We undertook clonogenic survival assays using small molecule inhibitors of the kinases ATR and ATM to compare the respective IR-induced cell killing by inhibition of ATR and ATM. ETP-46464 and KU55933 were used at 10 μM, a concentration leading to maximal respective inhibition of ATR or ATM (Supplementary Figure S1A). Surprisingly, we observed that a 4 h inhibition of ATR kinase activity with ETP-46464, from −15 min to +4 h post IR, caused significantly more IR-induced cell killing than ATM kinase inhibition with KU55933 over the same interval in U2OS, H460, 201 T, 239 T and Calu6 cells (Figure 1D, Supplementary Figure S1B and Supplementary Table S1). Similar results were obtained when ATR kinase activity was inhibited using Vertex compound 45, confirming the specificity of the radiosensitization by ATR inhibition (Supplementary Figure S1C). Concurrent inhibition of both ATR and ATM kinase activity radiosensitized cells still further, suggesting that the ATR and ATM kinase-dependent mechanisms of radioprotection are at least partially independent during this 4 h interval.

Because increased cell killing could be due to unrepaired DNA damage, we quantitated IR-induced DNA damage foci in cells treated with or without ATR kinase inhibitor. Transient ATR kinase inhibition caused an increase in the number of cells containing >50 RPA foci assayed 24 h after 2 or 10 Gy IR (Figure 1E), suggesting that the repair of some DNA lesion is impaired by ATR kinase inhibition resulting in the accumulation of regions of ssDNA that are coated with RPA.

### Transient inhibition of ATR after IR has long-term effects on DNA damage signalling

**Figure 2.**
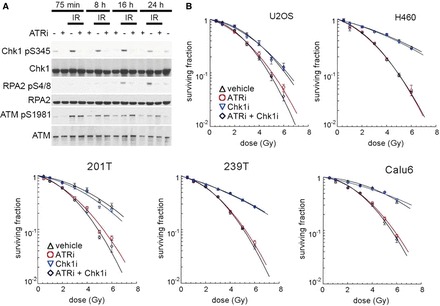
ATR inhibition causes long-term defects in the DNA damage response. (**A**) ATR inhibition for just 1 h (+15 to +75 min following IR) causes long-term defects in the DNA damage response. Immunoblots of lysates collected at indicated time points from U2OS cells that were mock-treated or treated with 2 Gy of IR and/or ETP-46464. (**B**) Chk1 inhibition for 1 h (+15 to +75 min following IR) does not cause the same effect as ATR inhibition. Immunoblots of lysates collected at indicated time points from U2OS cells that were mock-treated or treated with 2 Gy of IR and/or UCN-01. Clonogenic survival assays of Chk1 and/or ATR inhibitor-treated cells. Cell lines U2OS, H460, 201 T, 239 T and Calu6 were treated for 1 h with 100 nM UCN-01 and/or 10 µM ETP-46464. 177 × 236 mm (300 × 300 DPI).

ATR inhibitors increased IR-induced cell killing, even when cells were treated with them for only 1 h, from +15 to +75 min post IR (Supplementary Figure S2A and B). Significant aspects of ATR kinase signalling are mediated by the downstream Chk1 kinase (19). To determine whether abrogated ATR kinase-dependent Chk1 phosphorylation was associated with the radiosensitization caused by transient ATR kinase inhibition, exponentially growing U2OS cells were exposed to 2 Gy IR and treated with ETP-46464 for 1 hour (+15 to +75 min post IR). ATR kinase-dependent signalling to Chk1, as measured by S345 phosphorylation, was determined at 75 min, 8 h, 16 h and 24 h after IR. ATR kinase inhibition for just 1 hour suppressed Chk1 phosphorylation, and Chk1 phosphorylation was not recovered even 24 h after IR (Figure 2A). Although initial IR-induced ATM phosphorylation was not inhibited in cells treated with ATR kinase inhibitor, phosphorylated ATM levels were reduced in vehicle-treated cells relative to ETP-46464-treated cells at later time points (Figure 2A). The persistently high phospho-ATM levels suggest that DNA repair may be impeded in cells treated with ATR kinase inhibitor. Consistent with this hypothesis, high levels of phosphorylated RPA2 S4/8, a marker of extensive DNA end resection (20), were not detected in cells treated with ATR kinase inhibitor (Figure 2A). Furthermore, the absence of detectable phosphorylated RPA2 S4/8 in the first 8 h shows that the ATR kinase-dependent phosphorylation of Chk1 following IR can occur in the absence of extensive end resection. This finding is consistent with a recent report that ATR kinase-dependent phosphorylation of Chk1 following DNA damage does not require extensive end resection (8). The present data show that transient ATR kinase inhibition for just 1 h suppresses RPA phosphorylation at later time points, and by inference also inhibits extensive end resection. The ATR-dependent DNA damage repair leading to end resection appears to be governed by strict temporal constraints.

### Inhibition of Chk1 does not phenocopy inhibition of ATR

To determine whether abrogated Chk1 kinase signalling was associated with the radiosensitization, exponentially growing U2OS cells were exposed to 2 Gy IR and treated with the Chk1 kinase inhibitor UCN-01 (21) for 1 h (+15 to +75 min post IR). Chk1 kinase-dependent signalling to CDC25C, as measured by S216 phosphorylation, was determined at 75 min, 8 h, 16 h and 24 h after IR. While Chk1 kinase-dependent CDC25C phosphorylation was observed at the 75 min time point, no defect in CDC25C levels or serine-216 phosphorylation at 8 h, 16 h or 24 h were observed suggesting that transient inhibition of Chk1 kinase activity for 1 h after IR has no long-term consequences on CDC25C (Supplementary Figure S2C). To investigate the contribution of ATR kinase-dependent Chk1 signalling to radioprotection, we undertook clonogenic survival assays using ETP-46464 and UCN-01. Chk1 kinase inhibition for 1 h did not increase IR-induced cell killing in U2OS, H460, 201 T, 239 T or Calu6 (Figure 2B). Therefore, radiosensitization by ATR kinase inhibition can not be merely attributed to the lack of signalling through Chk1 during the 1 h interval.

### ATR kinase activity contributes to IR resistance in G1 phase cells

**Figure 3.**
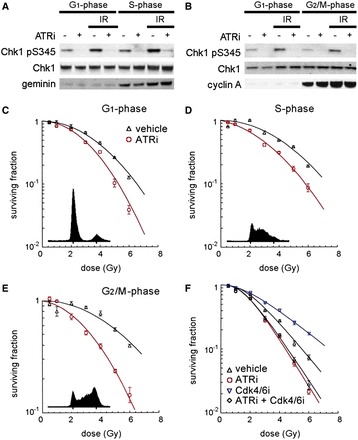
ATR inhibition radiosensitizes cells at all stages of the cell cycle. (**A** and **B**) ATR phosphorylates Chk1 after IR in G1. Immunoblots of synchronized U2OS following 2 Gy and/or ATR inhibition. U2OS in G1, S (A) or G2/M phase (B) were irradiated and treated with 10 µM ETP-46464 for 1 h (+15 to +75 min following IR) or vehicle before harvesting at +75 min. (**C–E**) Clonogenic survival assays of vehicle or ETP-46464 treated U2OS cells synchronized in G1 phase (C), S phase (D) or G2/M-phase (E). (**F**) Clonogenic survival assays of U2OS cells pre-treated for 24 h with the CDK inhibitor PD0332991. 139 × 193 mm (300 × 300 DPI).

To determine whether ATR contributes to radioresistance in G1 phase, we synchronized U2OS by subsequent blocks with thymidine and nocodazole (Supplementary Figure S3) and exposed G1, S and G2/M phase populations to 2 Gy IR. Synchronized cells were treated with ETP-46464 from +15 min to +75 min post IR, at which point whole cell lysates were prepared. Strong IR-induced ATR kinase-dependent phosphorylation of Chk1 was observed in S, G2/M and G1 phase cells showing that ATR kinase is functional in G1 phase (Figure 3A and B). Although S phase cells seem to have a higher base line level of phosphorylated Chk1 S345, the extent of IR-induced Chk1 phosphorylation was similar throughout the cell cycle.

Next, we investigated the cell cycle dependency of radiosensitization by ETP-46464 using synchronized U2OS. ATR inhibition increased IR-induced cell killing in S, G2/M and G1 phase cells, showing that in all phases of the cell cycle ATR kinase activity during the 1 h interval following exposure to IR is important for survival (Figure 3C–E).

CDK4/6 inhibition by the small molecule PD0332991 causes a reversible G1 arrest that has previously been associated with radiation protection in both human fibroblasts and mice (22). To determine whether CDK4/6 inhibition, and thus transient G1 arrest, would decrease the radiosensitization caused by transient ATR kinase inhibition, we undertook clonogenic survival assays using ETP-46464 and PD0332991. We found that CDK4/6 inhibition by PD0332991 from 24 h prior to 4 h post IR radioprotects U2OS cells (Figure 3F). However, PD0332991 treatment did not radioprotect cells that were transiently treated with ATR kinase inhibitor. These data show that the long-term effects of transient ATR inhibition cannot be reversed by transiently arresting cells in G1 phase. This result excludes cell cycle checkpoint defects and DNA replication-associated damage as the original mechanisms underlying radiosensitization.

### ATR forms foci in G1 phase cells following IR

**Figure 4.**
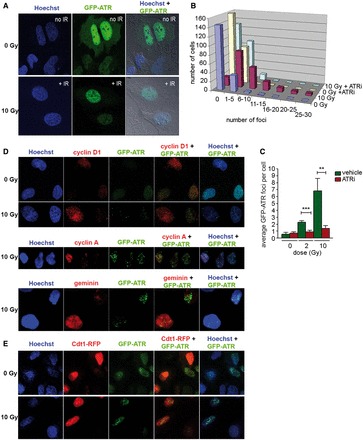
ATR has a role in the DNA damage response in G1 phase. (**A**) GFP-ATR forms foci after IR in G1. Images of synchronized U2OS cells stably expressing GFP-ATR were taken 75 min after 10 Gy IR. (**B**) Histogram of the cellular distribution of ATR foci in U2OS cells in G1 treated with 10 Gy IR. U2OS cells in G1 phase were treated for 1 h (+15 to +75 min following IR) with or without ETP-46464. ATR foci were quantitated 75 min following IR. (**C**) IR-induced ATR foci formation is ATR kinase-dependent. GFP-ATR expressing U2OS cells in G1 phase were treated for 1 h (+15 to +75 min following IR) with or without ETP-46464. GFP-ATR foci were quantitated 75 min following IR. Mean GFP-ATR foci per cell were enumerated in three independent experiments. Error bars denote standard deviations. (**D**) Images of asynchronous U2OS cells stably expressing GFP-ATR taken 75 min after 10 Gy IR. Cells were immunostained with antibodies to cyclin D1, cyclin A or geminin. (**E**) Images of U2OS cells expressing GFP-ATR and Cdt1-RFP taken 75 min after 10 Gy or mock treatment. 202 × 280 mm (300 × 300 DPI).

GFP-tagged ATR has been shown to form foci in U2OS cells in S and G2 phase after IR (5,23). We examined IR-induced foci in synchronized U2OS cells stably expressing GFP-ATR. A greater than 10-fold induction in the mean number of GFP-ATR foci was observed in G1 phase cells exposed to 2 or 10 Gy IR (Figure 4A–C). To confirm that IR-induced GFP-ATR foci form in G1 phase, we fixed asynchronous cells 1 h following 10 Gy IR and visualized geminin, cyclin A or cyclin D1 as well as GFP-ATR. While high geminin or cyclin A levels are markers for S and G2 phase, cyclin D1 levels are high in G1 phase, but low in S phase (24). We observed GFP-ATR foci in all U2OS cells regardless of positive or negative staining for each cell cycle marker (Figure 4D, for additional negative controls see Supplementary Figure S4A), showing that IR-induced GFP-ATR foci form in all phases of the cell cycle.

Cdt1 promotes the assembly of the pre-replication complex that is required for the loading of the DNA replication machinery. Cdt1 accumulates during G1 phase and is degraded in early S phase by ubiquitin-mediated proteolysis (25,26). We used a virus expressing a red fluorescent fusion protein, Cdt1-RFP, whose expression is restricted to G1 phase cells by the same mechanism as the endogenous protein, to investigate the formation of IR-induced ATR foci in G1 phase cells. We transduced an asynchronous population of U2OS cells that stably expresses GFP-ATR, exposed the cells to 10 Gy IR and fixed them after 1 h or monitored them by live cell microscopy for one day. IR-induced GFP-ATR foci formed in both Cdt1-RFP positive and negative cells (Figure 4E). Furthermore, live cell imagining of cells with GFP-ATR foci revealed a population of cells that decreased Cdt1-RFP expression in an interval of 2–8 h post IR (see movies in Supplementary Information). This is important because it excludes the possibility that the observed GFP-ATR foci in these Cdt1-RFP positive cells result from IR-induced damage in mitotic cells or appeared at the onset of S phase.

### ATR foci in G1 phase colocalize with RPA foci and their formation is ATR kinase-dependent

To determine whether the formation of IR-induced ATR foci is ATR kinase-dependent, we examined GFP-ATR foci in cells 75 min post IR. ATR kinase inhibition from +15 to +75 min post IR strongly inhibited the formation of ATR foci in both synchronized G1 phase cells (Figure 4B and C) and asynchronous cells (Supplementary Figure S4B). Thus, ATR kinase activity is required for the formation or stability of IR-induced ATR foci, indicating that phosphorylation of a yet to be identified target within 1 h after IR is an important step in the DNA damage signalling.

**Figure 5.**
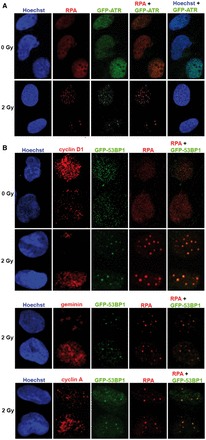
RPA colocalizes with 53BP1 and ATR in G1 phase. (**A**) IR-induced ATR foci colocalize with RPA foci. GFP-ATR expressing U2OS cells in G1 phase were treated with 2 Gy IR and fixed after 75 min. Cells were immunostained with antibodies against RPA2. (**B**) Asynchronous GFP-53BP1 expressing U2OS cells were treated with 2 Gy IR and fixed after 75 min. Cells were co-immunostained with antibodies against RPA2 (detected by anti-mouse-Alexafluor647) and the cell cycle markers cyclin D1, geminin or cyclin A (detected by anti-rabbit-Cy3). 134 × 287 mm (300 × 300 DPI).

**Figure 6.**
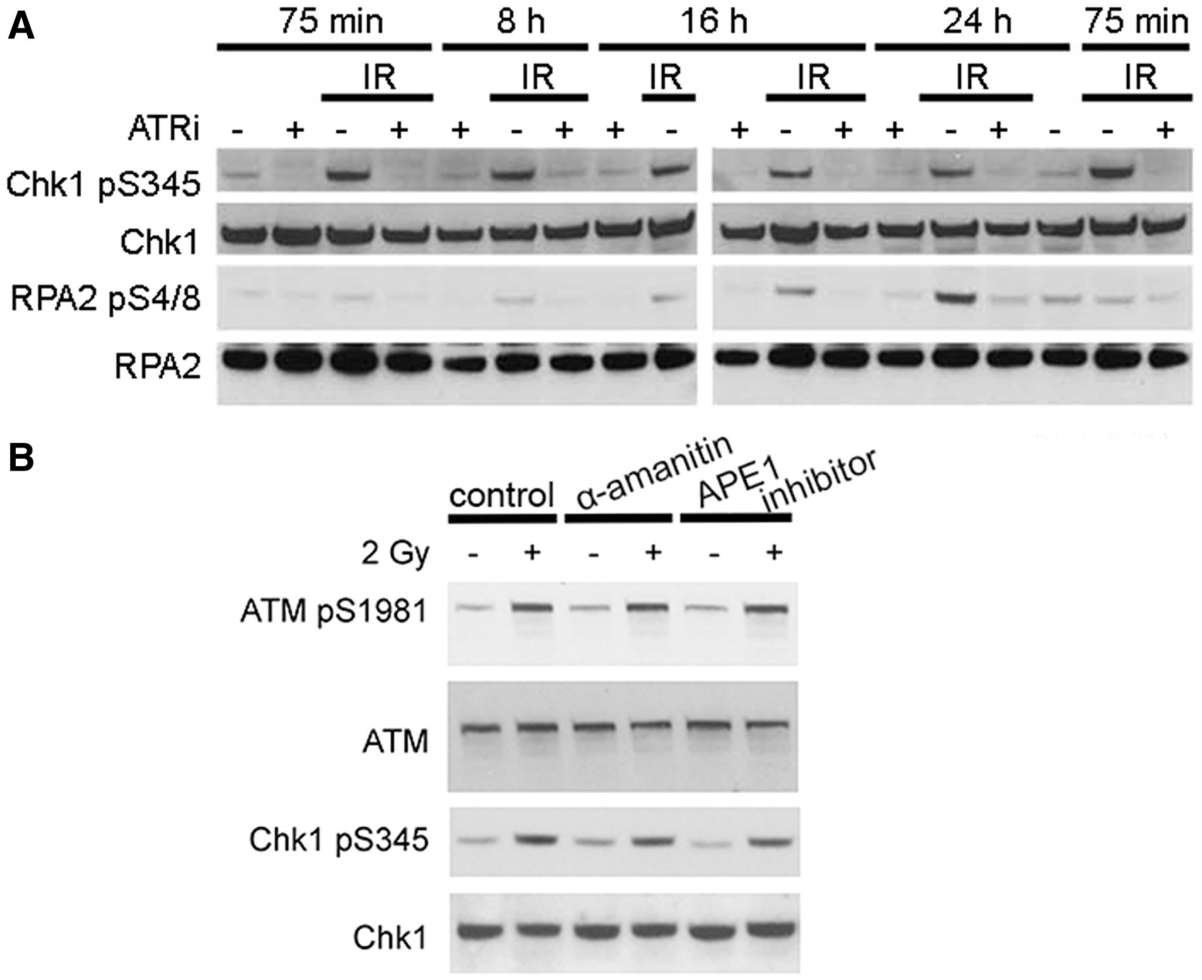
Chk1 phosphorylation by ATR in G1 phase occurs in the absence of RPA2 phosphorylation and is independent of transcription. (**A**) Immunoblots of synchronized U2OS irradiated in G1 phase with 2 Gy and/or treated with ETP-46464 for 1 h (+15 to +75 min following IR). (**B**) Immunoblots of synchronized U2OS following 2 Gy and/or inhibition of transcription or BER. Synchronized U2OS cells in G1 were treated for 1 h with an inhibitor of RNA polymerases II and III (α-amanitin) or of APE1 (NSC332395) before exposure to 2 Gy IR. Inhibitors were not taken off and cells were harvested 1 h after IR. 127 × 122 mm (300 × 300 DPI).

ATR recruitment to RPA-coated ssDNA via its binding partner ATRIP is the initial step required for DNA damage-induced ATR kinase activation (27,28). Previous models proposed the need of extensive DNA end resection after IR resulting from HDR repair to activate ATR (5). Because HDR is generally not associated with G1 phase, we wanted to see whether RPA foci can form in G1 phase and whether they are the site of ATR recruitment. Synchronized U2OS cells (6 h after nocodazole release) stably expressing GFP-ATR were exposed to 2 Gy IR and fixed after 1 h. Confocal microscopy showed that IR could induce RPA foci in G1 phase and that all ATR foci colocalized to RPA foci (Figure 5A), further confirming that HDR is not needed for ATR activation. Of note, RPA foci were observed just 1 h after IR, long before detectable RPA2 S4/8 phosphorylation (Figures 2A and 6A).

### 53BP1 colocalizes with RPA foci in irradiated G1 phase cells

RPA foci in G1 phase cells have recently been reported also in DT40 chicken cells (29). Previous models have suggested that the extensive end resection associated with HDR repair of IR-induced DSBs is required to generate the RPA-coated ssDNA that recruits ATR after IR (5). Ionizing radiation causes not only DSBs but also other types of DNA damage including single-strand breaks. To see whether the observed RPA foci in G1 could be at DSBs, we investigated the incidence of RPA foci in U2OS cells stably expressing GFP-53BP1 irradiated in G1 phase. Asynchronous cells were exposed to 2 Gy IR, fixed after 1 h, and RPA2 and the cell cycle markers cyclin D1, cyclin A, or geminin as well as GFP-53BP1 were visualized. The majority of RPA foci colocalized with GFP-53BP1 (Figure 5B, for additional negative controls see Supplementary Figure S5). Furthermore, IR-induced RPA foci were evident in all U2OS cells regardless of positive or negative staining for geminin, cyclin A or cyclin D1 (Figure 5B) and the colocalization of RPA foci with GFP-53BP1 was not cell cycle-dependent.

## DISCUSSION

The use of small molecule inhibitors to transiently inhibit the kinase activity proved absolutely critical to determine the role of the essential protein ATR following an acute event such as irradiation and to perform a temporal analysis of its function. Our findings that ATR kinase activity has an important role in G1 phase cells and that ATR inhibition for a short clinically achievable time after IR enhanced cytotoxicity are significant and have great translational potential. It is estimated that 50–60% of cancer patients receive radiation therapy (30). Previous reports have focused on the essential functions of ATR kinase activity in S and G2 phase, and this may have obscured the potential role of ATR kinase inhibitors as radiosensitizers because at any given time the majority of cancer cells will be in G1 phase, especially *in vivo*. Our data indicate that ATR inhibitors promise to be excellent drugs for improving radiation therapy and illuminate a new facet in the DNA damage repair.

### Activation of ATR in G1 phase

Our experiments unambiguously show that ATR kinase is activated in irradiated G1 phase cells: ATR forms foci and phosphorylates Chk1 in irradiated G1 phase cells. Furthermore, inhibition of ATR in irradiated G1 phase cells increases cell killing, indicating an important role of ATR in the DNA damage signalling also in G1 phase. ATR kinase activation in irradiated G1 phase cells challenges the current dogma that restricts ATR kinase activation to RPA-coated ssDNA generated at aberrant replicative structures and resected DSBs that are generated during HDR mechanisms in S and G2 phase cells. Furthermore, we observe ATR-dependent Chk1 phosphorylation in irradiated G1 phase cells long before detectable RPA2 phosphorylation (Figure 6A), which is regarded as a marker of extensive end resection (20). Previous models suggested that ATR activation requires CtIP-mediated end resection and that without CDK-mediated phosphorylation of CtIP in S and G2 phase ATR cannot be activated (7). However, a recent report showed that CtIP activity is dispensable for Chk1 phosphorylation, but plays a role in the check point maintenance (8). Our finding that ATR activation after IR precedes RPA2 S4/8 phosphorylation is consistent with this report and extends it insofar as we observe ATR activation in irradiated G1 phase cells.

### RPA-coated DNA in G1 phase after IR

Our observation that IR-induced ATR foci colocalize with RPA foci in G1 phase cells suggests that ATR is activated by the canonical recruitment of ATR/ATRIP to RPA-coated ssDNA. In this regard, RPA foci in G1 phase cells have recently been reported in DT40 chicken cells (29). The exact origin and structure of the RPA foci/ssDNA we observed in G1 phase are presently unknown, but considering that the structure is not a result of HDR (or replication stall), the DNA might not have the 5′-recessed junction, which is needed for loading of the 9-1-1 complex (31,32). TopBP1 is an allosteric activator of ATR (33,34). It is possible that in contrast to the damage induced by replication stress (35,36), TopBP1 recruitment to damage in G1 phase may not require the 9-1-1 complex. It is interesting that TopBP1 has been reported to colocalize with DSBs specifically in G1 phase (37).

The ssDNA in G1 phase could result from DNA damage at actively transcribed sites or during base excision repair (BER). First, we tested whether ATR kinase activity in G1 phase was transcription-dependent by inhibiting RNA polymerases II and III with a high dose of α-amanitin (50 µg/ml) in synchronized U2OS cells prior to IR. No significant reduction in IR-induced Chk1 phosphorylation was detected 1 h after 2 Gy IR when transcription was inhibited (Figure 6B). Next, we inhibited the APE1 that is required for BER-associated gap tailoring in synchronized U2OS cells prior to IR. No significant reduction in IR-induced Chk1 phosphorylation was detected 1 h after 2 Gy IR when BER was inhibited (Figure 6B). Nevertheless, the majority of IR-induced RPA foci colocalized with 53BP1 foci at 1 h post IR (Figure 5B), strongly suggesting that the colocalized RPA and ATR foci in G1 phase are at DSBs. We speculate that structures at IR-induced DSBs arising during the processing preceding the extensive end resection that is required for HDR are associated with sufficient RPA-coated ssDNA to activate ATR in G1 phase. Future experiments will determine whether this is a characteristic of fast growing cells, such as the studied cancer cells, or whether it is also found in non-transformed fibroblasts.

### A critical window for ATR activation after IR

We discovered that ATR inhibition during a short interval after IR is sufficient to not only significantly radiosensitize cells, but also has long-term effects on the DNA damage signalling. Remarkably, Chk1 and RPA2 phosphorylation are not recovered when ATR kinase is transiently inhibited for 1 h after IR (Figure 6A) despite the fact that the ATR kinase inhibitor is reversible within 30 min (Figure 1C). This is true for both irradiated asynchronous and synchronized G1 phase cells. Such a critical window for DNA damage response pathway activation is reminiscent of our previous studies that revealed transient ATM kinase inhibition from +15 to +75 min after IR to cause long-term consequences in cells that included an abrogation of sister chromatid exchange, an accumulation of chromatid breaks and increased cell death (38,39). We subsequently showed that ATM kinase activity plays an important role in the dynamic exchange of proteins in DSB repair complexes and hypothesized that ATM kinase inhibition disrupts the integration of multiple protein activities that must be spatially and temporally coordinated at sites of DSBs for appropriate repair (40,41). Our experiments show that transient inhibition of ATR or ATM kinase activities irreversibly disrupts DNA damage signalling pathways and/or assembled repair complexes. This reveals an unexpectedly strict temporal requirement for ATR and ATM kinase activities following DNA damage. This capacity to block repair by exposing cancer cells to a short treatment with drugs that target key components of the DNA damage signalling has significant clinical potential. Radiosensitization promises to reduce the exposure of patients to ionizing radiation and to thereby minimize harmful side effects such as damage to the tissue surrounding the tumor. Administration of ATR inhibitors to patients for just a brief interval would allow a decrease in drug toxicity that is not associated with radiation. Future experiments will determine whether ATR inhibition also radiosensitizes primary cells and whether the pharmacological properties of novel ATR inhibitors currently developed allow for a short drug exposure or even local administration to the patient.

## SUPPLEMENTARY DATA

Supplementary Data are available at NAR Online.

## FUNDING

Frieda G. and Saul F. Shapira BRCA Cancer Research Program (in part) and NIH Grants [RO1 CA148644, U01CA099168, U19-AI068021, U54GM103529, P30CA047904 and P50CA090440]; ETP-46464 and Vertex compound 45 were synthesized by the Medicinal Chemistry Shared Resource of the Ohio State University Comprehensive Cancer Center (Columbus, OH), the National Cancer Institute Grant [P30 CA16058] (in part). Funding for open access charge: [RO1 CA148644].

*Conflict of interest statement*. None declared.

## Supplementary Material

Supplementary Data
