# Supplementary material for: ATR kinase activation in G1 phase facilitates the repair of ionizing radiation-induced DNA damage
Source: Nucleic Acids Res. 2013 Sep 14;41(22):10334–44. doi: 10.1093/nar/gkt833 (PMC3905881; doi:10.1093/nar/gkt833)
Supplement: Supplementary Data [file supp_41_22_10334__index.html]

ATR kinase activation in G1 phase facilitates the repair of ionizing radiation-induced DNA damage — ATR kinase activation in G1 phase facilitates the repair of ionizing radiation-induced DNA damage — Supplementary Data 

# ATR kinase activation in G1 phase facilitates the repair of ionizing radiation-induced DNA damage

## Supplementary Data

files

**Files in this Data Supplement:**

- Supplementary Data - pdf file
- Supplementary Data - mp4 file
- Supplementary Data - mp4 file
