# Supplementary material for: ATR kinase activation in G1 phase facilitates the repair of ionizing radiation-induced DNA damage
Source: Nucleic Acids Res. 2013 Sep 14;41(22):10334–44. doi: 10.1093/nar/gkt833 (PMC3905881; doi:10.1093/nar/gkt833)
Supplement: Supplementary Data [file supp_gkt833_nar-01534-d-2013-File008.pdf]

ATR kinase activation in G1 phase facilitates the repair of ionizing radiation-induced DNA damage

Armin M. Gamper 1, Reza Rofougaran 1, Simon C. Watkins 2, Joel S. Greenberger 1, Jan H. Beumer 4, Christopher J. Bakkenist 1,3

1 Department of Radiation Oncology, 2 Department of Cell Biology and Physiology, Center for Biologic Imaging, and 3 Department of Pharmacology and Chemical Biology, University of Pittsburgh School of Medicine.
4 Department of Pharmaceutical Sciences, University of Pittsburgh School of Pharmacy.

Supplementary Table S1. ATR inhibition has a more potent radiosensitizing effect than ATM inhibition. (**A**) Clonogenic survival curves were fitted to a Linear Quadratic Model: $S=\exp(-(\alpha D+\beta D^2))$, with S = survival, D=dose. Calculated α and β values for cell lines U2OS, H460, 201T, 239T, and Calu6 are shown in Table S1A. (**B**) Sensitization of cell lines U2OS, H460, 201T, 239T, and Calu6 by 10 µM KU55933 or 10 µM ETP-46464. Values of the fitted curves were used to calculate the sensitization at 4 and 5 Gy. Table S1B shows average sensitization at 4 and 5 Gy in the various cell lines and their standard deviation.

A

		alpha no inhib	beta no inhib	alpha ATM inh	beta ATM inh	alpha ATR inhib	beta ATR inhib	alpha ATM+ATR inh	beta ATM+ATR inh
U2OS	1st	0.03566	0.03698	0.07468	0.05869	0.3182	0.04559	0.1465	0.1318
	2nd	0.05836	0.05083	0.169	0.06247	0.7381	0	0.4933	0.08505
	3rd	0.05731	0.03579	0.1178	0.05402	0.2537	0.06989	0.3001	0.1213
H460	1st	0.08844	0.01726	0.08993	0.06415	0.4319	0.03482	0.5669	0.07285
	2nd	0.306	0.007229	0.08375	0.09814	0.8924	0	1.025	0.05351
	3rd	0	0.04366	0.06962	0.04912	0.408	0.07758	0.5911	0.05914
239T	1st	0.05765	0.03131	0.2123	0.07319	0.5849	0.04853	1.254	0
	2nd	0.06907	0.01561	0.2886	0.01682	0.2243	0.1013	0.7145	0.06131
	3rd	0.03296	0.02076	0.1747	0.03265	0.1758	0.1039	0.7147	0.07147
201T	1st	0.1202	0.01221	0.07234	0.06343	0.3108	0.07781	0.5004	0.07231
	2nd	0.1289	0.01277	0.06747	0.06251	0.3213	0.08057	0.4697	0.1237
	3rd	0.1655	0.01128	0.07693	0.06196	0.3605	0.05314	0.4778	0.09616
Calu6	1st	0	0.01758	0.0428	0.04059	0.07696	0.07168	0.3366	0.1697
	2nd	0.05592	0.01382	0.06625	0.04366	0.1402	0.04948	0.1544	0.1256
	3rd	0.08501	0.02161	0.02423	0.08537	0.2042	0.1368	0.5821	0.0866

B

		4 Gy<br>no inhib	5 Gy<br>no inhib	4 Gy<br>ATM inh	5 Gy<br>ATM inh	4 Gy<br>ATR inhib	5 Gy<br>ATR inhib	4 Gy<br>ATM+ATR inh	5 Gy<br>ATM+ATR inh
U2OS	average	1	1	1.7450	2.1840	4.6876	6.5842	9.1083	22.6271
	stdev	0	0	0.1157	0.1249	1.7673	1.6756	1.7567	5.2341
H460	average	1	1	1.7775	2.6906	7.7770	14.0864	22.4443	62.6714
	stdev	0	0	0.3445	0.9246	2.2181	4.8550	12.8652	46.6844
239T	average	1	1	2.7379	3.9990	8.2934	18.7696	44.8121	122.4984
	stdev	0	0	0.7874	1.8914	2.2399	2.5832	24.2816	52.6884
201T	average	1	1	1.7287	2.5544	5.5909	11.7243	16.1972	52.6156
	stdev	0	0	0.1477	0.2764	1.1584	3.6423	5.9960	31.0199
Calu6	average	1	1	1.8566	2.6851	5.2951	13.9065	24.4525	109.6696
	stdev	0	0	0.2763	0.8211	4.2417	15.9772	17.7871	115.2622

Supplemental Figure legends

Supplementary Figure S1. ATR inhibition has a more potent radiosensitizing effect than ATM inhibition. (**A**) 10 µM ETP-46464 or 10 µM KU55933 maximally inhibit ATR or ATM respectively. Cell lines U2OS, H460, 201T, and 239T were treated with 2 or 5 Gy and the indicated concentration of ATR or ATM inhibitor. Immunoblots of lysates prepared 75 min following IR are shown for ETP-46464 and KU55933. (**B**) Sensitization of cell lines U2OS, H460, 201T, 239T, and Calu6 by 10 µM ETP-46464 or 10 µM KU55933. Clonogenic survival curves were fitted to a Linear Quadratic Model: $S=\exp(-(\alpha D+\beta D^2))$, with S = survival, D=dose. (see Supplementary Table 1). Values of the fitted curves were used to calculate the sensitization at 4 and 5 Gy. Graphs show average sensitization at 4 and 5 Gy in the various cell lines and their standard deviation. A Student's t-test was done for a two-tailed distribution of two samples with unequal variances. *, **, and *** denote p values smaller than 0.1, 0.05, or 0.01 respectively. (**C**) Clonogenic survival assays of vehicle, KU55933 and/or Vertex Compound 45 treated cells. Cell lines U2OS, 201T, 239T, Calu6 and H460 were treated for 4.25 h (15 min prior to 4 h post IR) with 10 µM KU55933 and/or 10 µM Vertex Compound 45.

Supplementary Figure S2. Chk1 inhibition for 1 h (+15 to +75 min following IR) does not cause the same effect as ATR inhibition. (**A**, **B**) ATR1 h ATR inhibition post IR is sufficient to radiosensitize cells. Clonogenic survival assays of ATR inhibitor-treated cells. U2OS cells were treated for 1 h (+15 to +75 min following IR) with 10 µM ETP-46464 (A) or 10 µM Vertex compound 45 (B). (**C**) Immunoblots of lysates collected at indicated time points from U2OS cells that were mock-treated or treated with 2 Gy of IR and/or the Chk1 inhibitor UCN-01.

Supplementary Figure S3. Schematic of the cell synchronization protocol, including FACS profiles, and the protocol for clonogenic survival assays with ATR inhibition from 15 to 75 min post irradiation.

Supplementary Figure S4. ATR has a role in the DNA damage response in G1 phase and ATR kinase activity is needed for foci formation or stability. (**A**) Negative controls for Figure 4D. Images of mock-treated, GFP-ATR expressing U2OS cells (0 Gy) immunostained with antibodies to Cyclin A or geminin. (**B**) Normalized increase in GFP-ATR foci per cell in asynchronous U2OS cells following IR. GFP-ATR foci 75 min after IR of asynchronous U2OS cells treated for 1 h (+15 to +75 min following IR) with 10 µM ETP-46464 were quantitated. The average of GFP-ATR foci per cell were enumerated in 3 independent experiments. Error bars denote standard deviations.

Supplementary Figure S5. RPA colocalizes with 53BP1 in G1 phase. Negative controls for Figure 5B. Asynchronous GFP-53BP1 expressing U2OS cells were treated with 2 Gy IR and fixed after 75 min. Cells were co-immunostained with antibodies against RPA2 (detected by anti-mouse-Alexafluor647) and the cell cycle markers geminin or cyclin A (detected by anti-rabbit-Cy3).

Supplementary Movies. Live cell imagining of cells with GFP-ATR foci. Movies show U2OS cells stably expressing GFP-ATR that were infected with a virus expressing Cdt1-RFP and irradiated with 10 Gy. The time portrayed by the movie corresponds to 24 h.

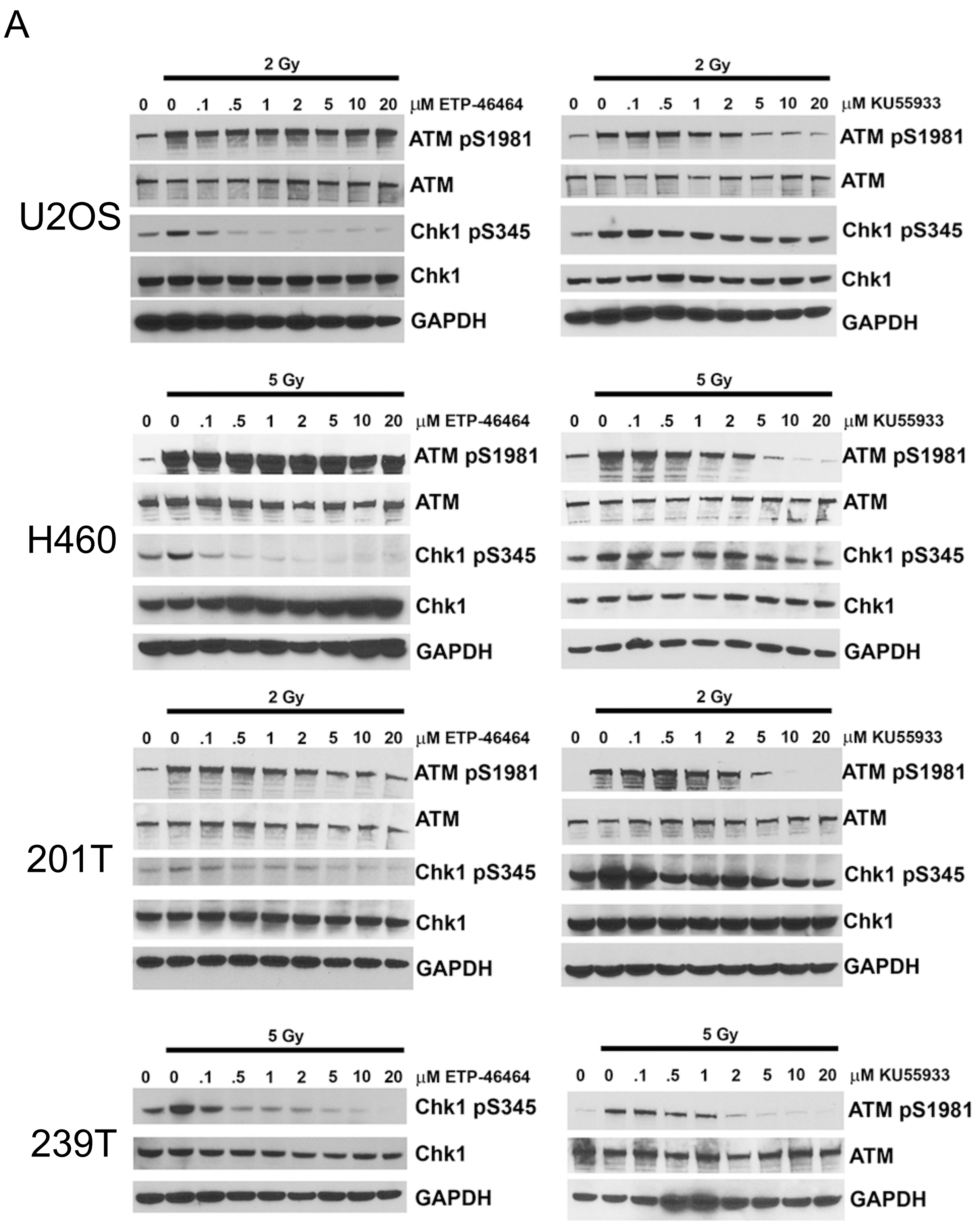

Gamper et al. Supplementary Figure S1A

B

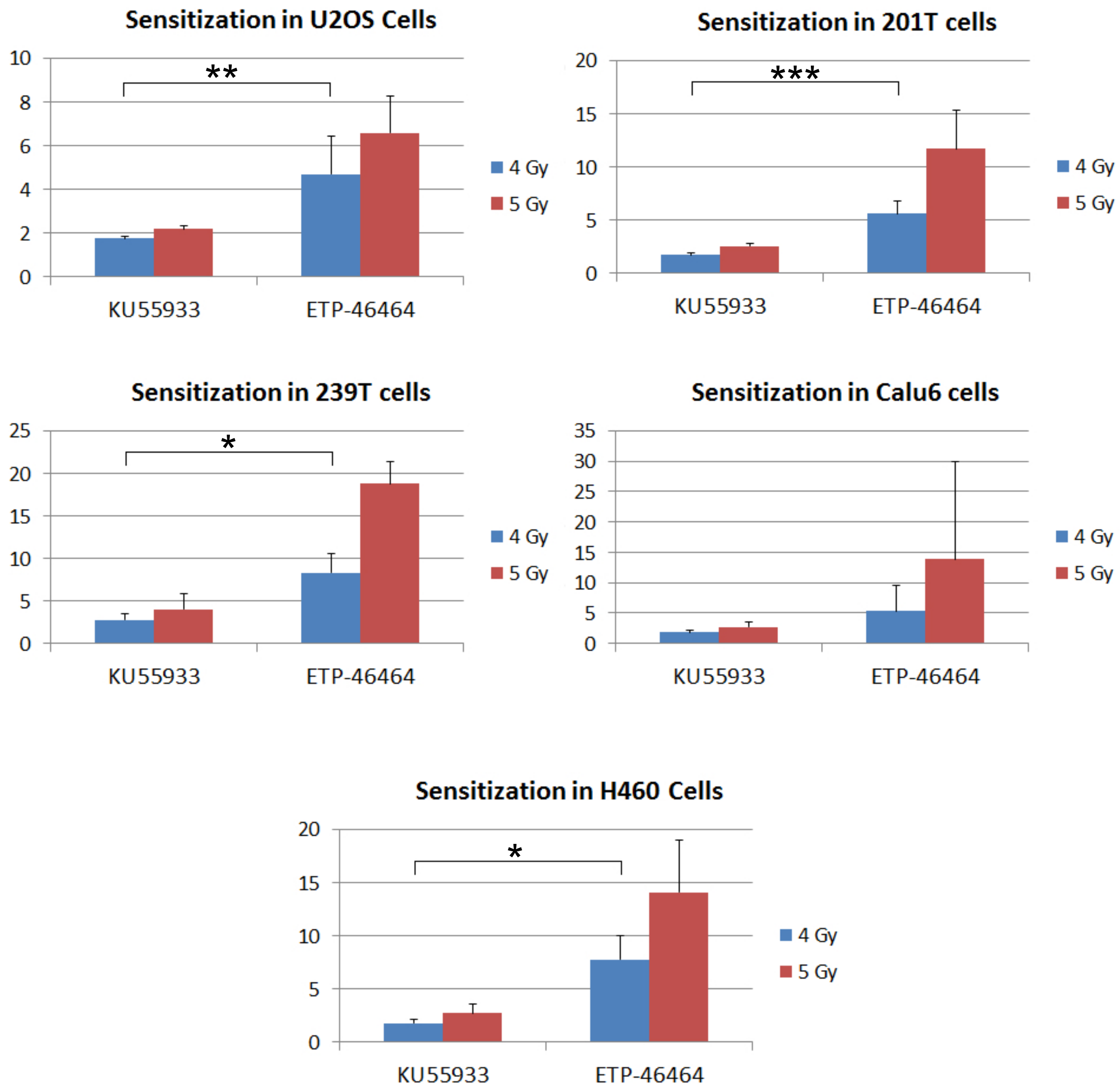

Gamper et al. Supplementary Figure S1B

C

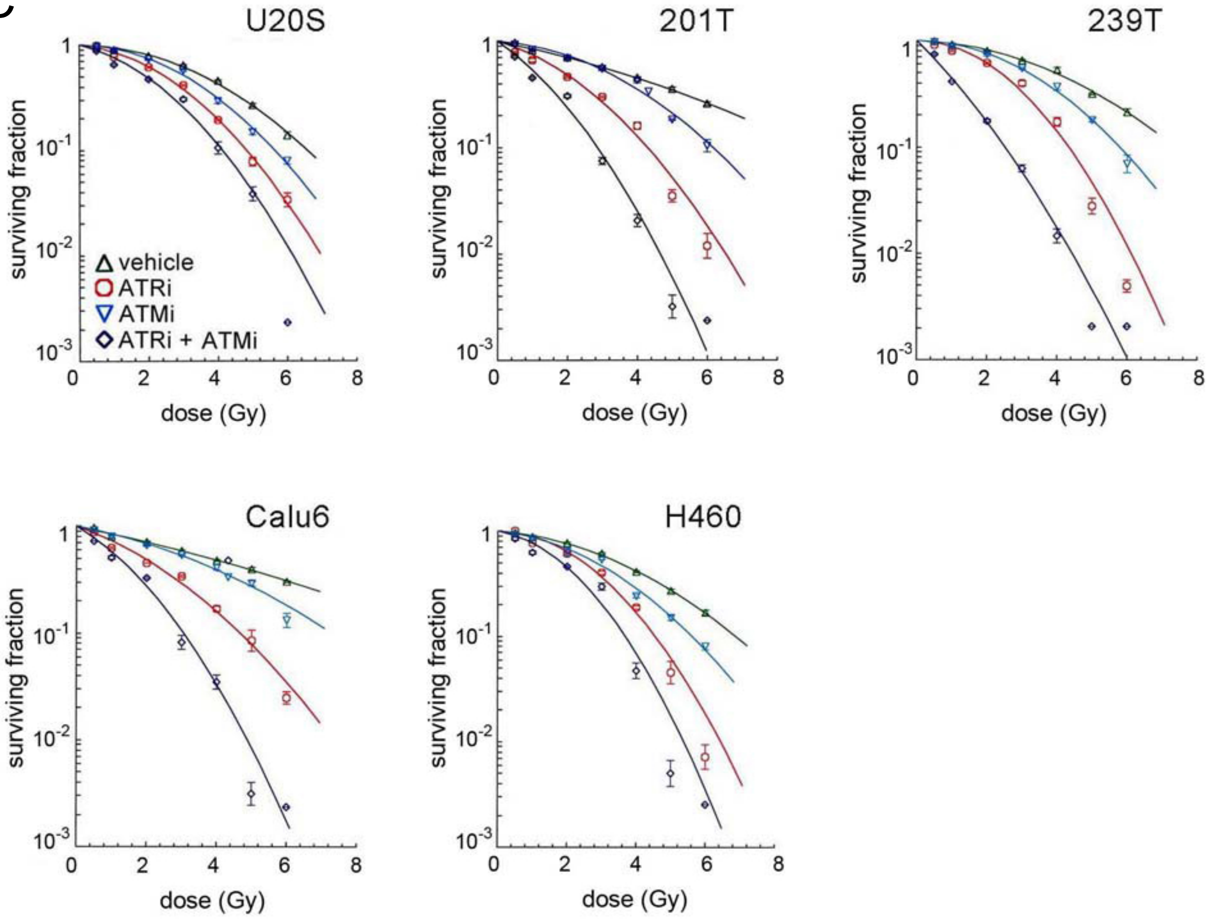

Gamper et al. Supplementary Figure S1C

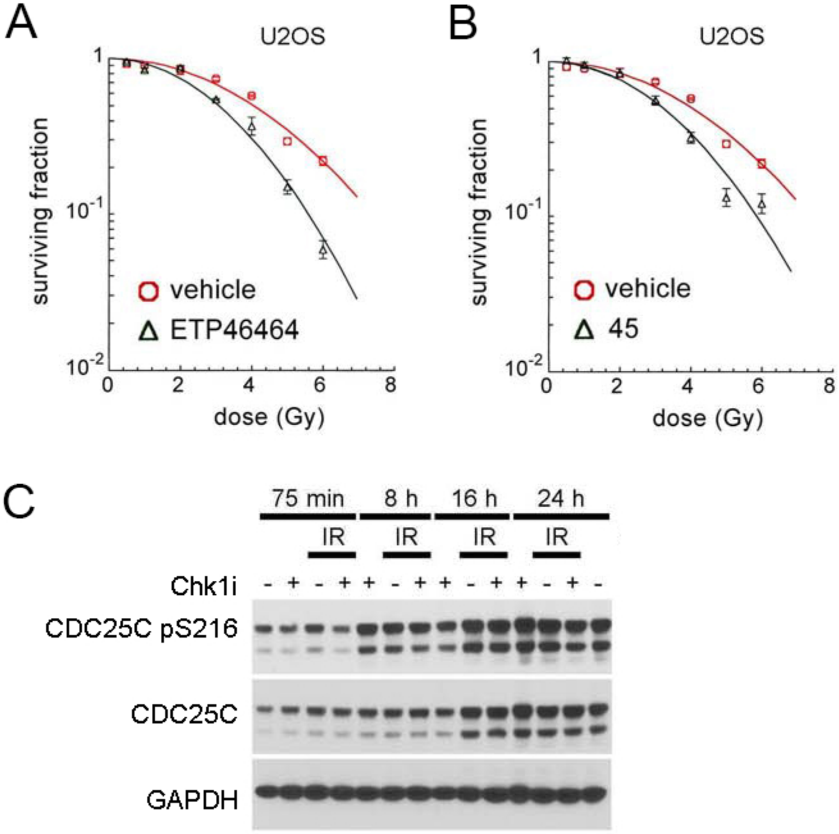

Gamper et al. Supplementary Figure S2

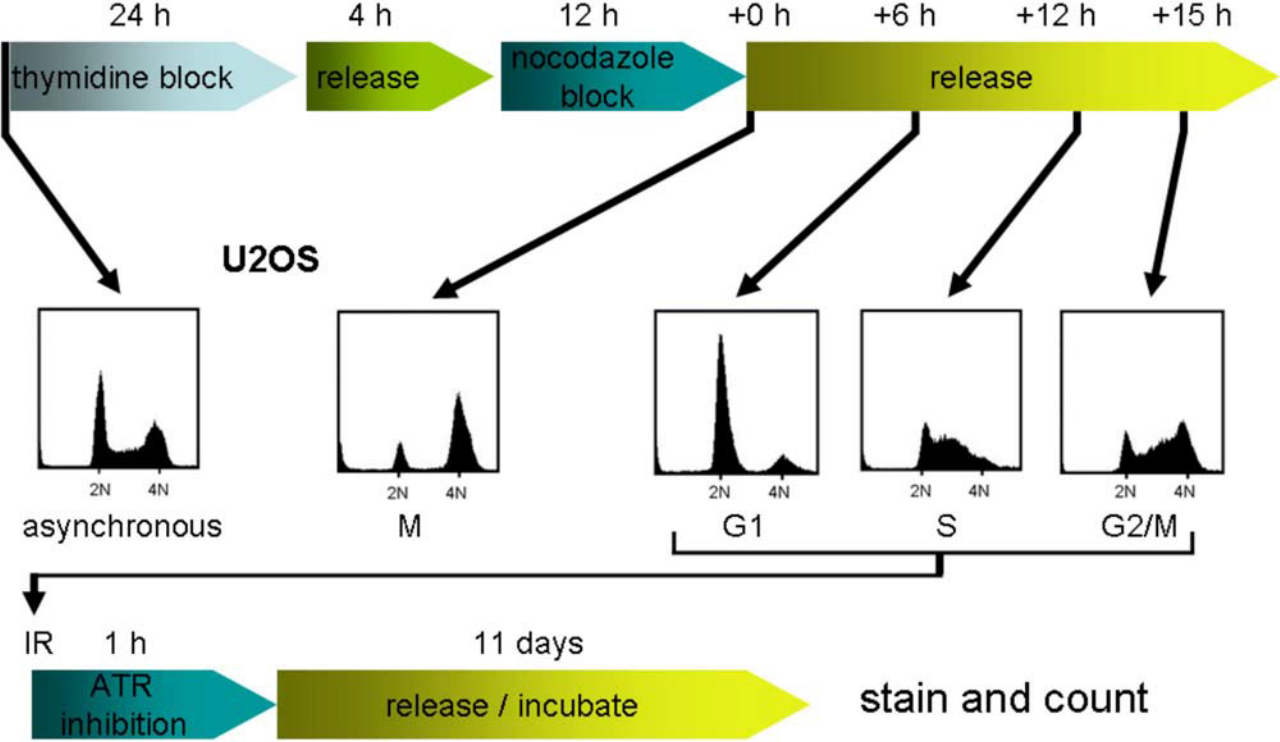

Gamper et al. Supplementary Figure S3

A

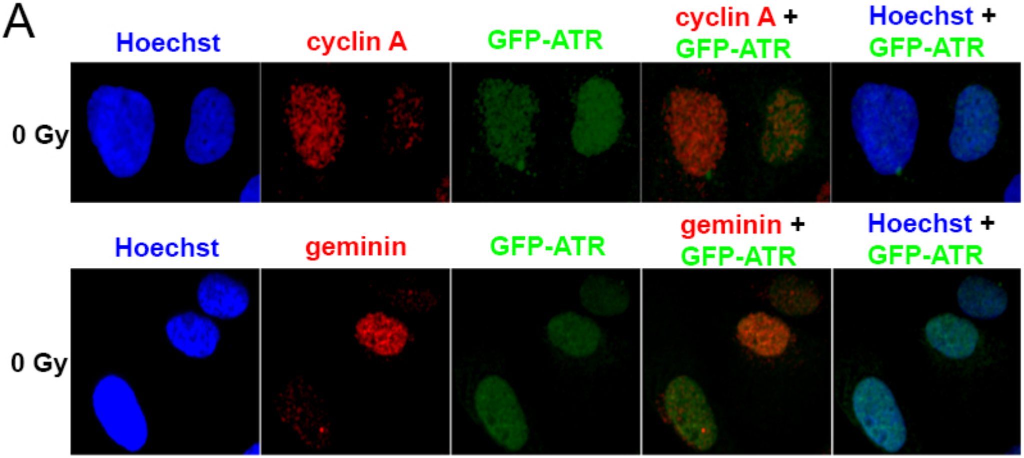

B

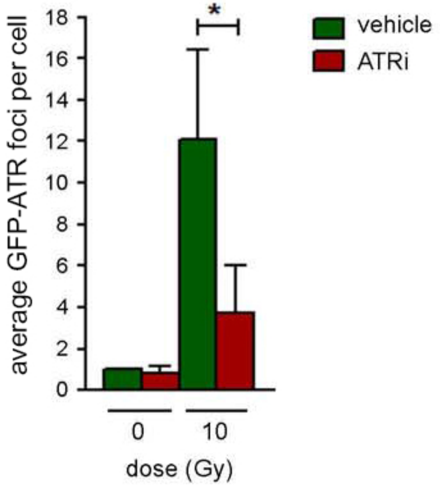

Gamper et al. Supplementary Figure S4

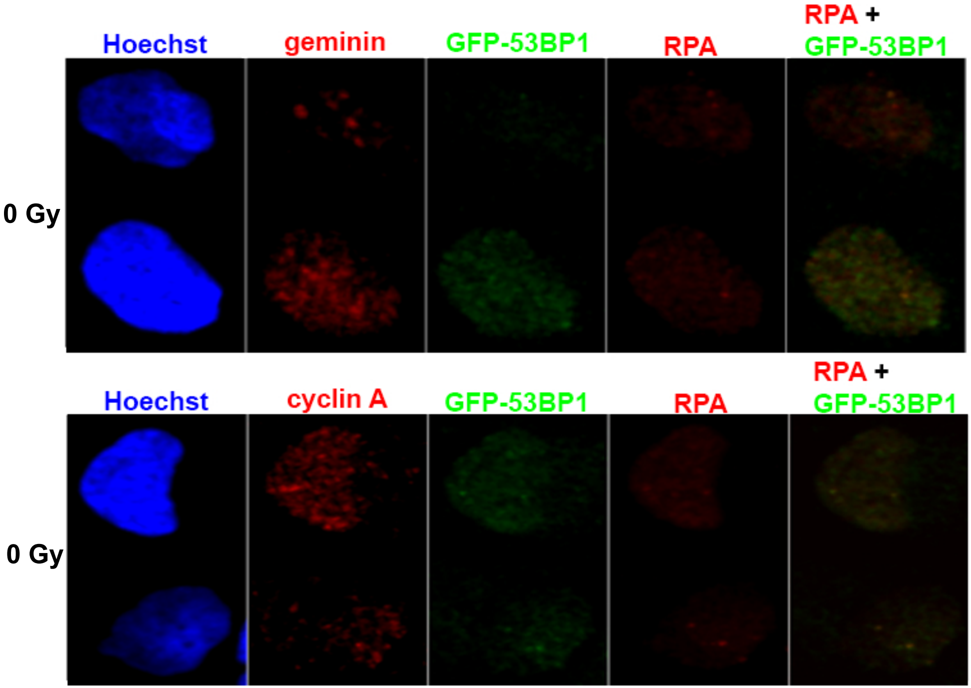

Gamper et al. Suppl. Fig. 5
